# The perirenal fat thickness was independently associated with serum uric acid level in patients with type 2 diabetes mellitus

**DOI:** 10.1186/s12902-022-01081-9

**Published:** 2022-08-20

**Authors:** Yuxian Yang, Yan Ma, Yanan Cheng, Yuechao Xu, Yuan Fang, Jing Ke, Dong Zhao

**Affiliations:** Center for Endocrine Metabolism and Immune Diseases, Beijing Luhe Hospital, Capital Medical University, Beijing, China

**Keywords:** Perirenal fat thickness, Paranephric fat thickness, Serum uric acid, Type 2 diabetes mellitus, Obesity

## Abstract

**Background:**

Obesity is an important risk factor for hyperuricemia. We aimed to explore the relationship between perirenal fat thickness (PrFT) and paranephric fat thickness (PnFT) and serum uric acid (SUA) in patients with type 2 diabetes mellitus (T2DM).

**Methods:**

This was a cross-sectional study involving 257 patients with T2DM recruited from Beijing Luhe Hospital from September 2019 to May 2020. The basic and clinical information such as age, gender, duration of diabetes was collected through the medical records. All patients underwent a physical examination including height, weight, waist circumference, hip circumference, systolic blood pressures and diastolic blood pressure. The venous blood and urine samples were collected to measure SUA, fasting blood glucose, total cholesterol, triglyceride, low-density lipoprotein-cholesterol, high-density lipoprotein-cholesterol, serum creatinine, blood urea nitrogen and glycosylated hemoglobin. PrFT and PnFT were measured via ultrasonography. Pearson correlation test and linear regression analysis were used to analyze the association between PrFT and PnFT and SUA.

**Results:**

We found that PrFT and PnFT increased according to the tertiles of SUA level (*P* = 0.001 and *P* = 0.009, respectively). In addition, the PrFT and PnFT were positively associated with SUA level (*r* = 0.25, *P* < 0.001, *r* = 0.23, *P* < 0.001, respectively). Moreover, this association was stronger in males, non-obesity patients and patients with normal renal function. In the multivariate analysis, the PrFT was independently associated with SUA level after adjusting confounding factors.

**Conclusions:**

The PrFT was independently associated with SUA level in patients with T2DM.

**Supplementary Information:**

The online version contains supplementary material available at 10.1186/s12902-022-01081-9.

## Introduction

Serum uric acid (SUA) is the end-product of purine metabolism. In recent years, the prevalence of hyperuricemia ranged from 9.3% to 37% in different countries around the world [1–5]. The overall prevalence of hyperuricemia in China was 14% [4]. Hyperuricemia was reported to be appeared in 32.6% of patients with type 2 diabetes mellitus (T2DM) [6, 7] and was an independent risk factor for the complications of T2DM, especially diabetic kidney diseases [8–11].

Obesity is an important risk factor for hyperuricemia [1, 12–16]. As a traditional indicator of general obesity, body mass index (BMI) was positively related to SUA level [1, 13, 14]. Furthermore, visceral fat tissue and waist circumference, have been reported to be more related to SUA than BMI [12, 15]. Perirenal fat and paranephric fat were ectopic visceral fat and were located around the kidney in the retroperitoneal space. Perirenal fat was wrapped by a complete renal fascia and had a complete system of blood supply, lymph fluid drainage and innervation. All these make perirenal fat like an internal organ. As a mixture of brown adipose tissue and white adipose tissue, perirenal fat can synthesis and secrete many adipokines, such as leptin, adiponectin and so on. While paranephric fat was different from perirenal fat anatomically and histologically. Paranephric fat, lied adjacent to perirenal fat, was not wrapped by renal fascia and was a typical white adipose tissue [17]. Multiple studies demonstrated that single perirenal fat was significantly associated with metabolic syndrome, renal function [18, 19] as well as atherosclerosis [20, 21]. Para-perirenal fat, which means the sum of perirenal fat and paranephric fat, was independently related to blood pressure [22] and reduced renal function [23].

Few studies explored the relationship between perirenal fat and paranephric fat and SUA level. Some cross-sectional studies have shown that perirenal fat thickness (PrFT) and paranephric fat thickness (PnFT) were both positively related to SUA in diabetic patients in univariate analysis [19, 24]. Another study reported that para-perirenal fat thickness was an independent predictor of SUA level in diabetic patients after adjustment for traditional metabolic factors, while did not distinguish PrFT from PnFT [25]. Therefore, it is still not clear whether PrFT or PnFT is independently associated with SUA level respectively. Therefore, we conducted this study to explore the relationship between PrFT and PnFT and SUA level patients with T2DM.

## Materials and methods

### Study population and study design

This was a cross-sectional study. A total of 257 inpatients with T2DM were recruited from Beijing Luhe Hospital from September 2019 to May 2020. As regards the criteria for inclusion, subjects enrolled were diagnosed with T2DM and were older than 18 years. Exclusion criteria included the presence of malignant tumor, present pregnancy, liver dysfunction, resistant hypertension, unstable angina, severe heart failure, severe elevated triglyceride (TG) and total cholesterol (TC) level. In addition, those who using anti-hyperuricemic agents and diuretics, undergoing major surgery, with elevated creatinine level and need renal replacement therapy (renal transplant or dialysis), with renal morphological abnormalities or low-quality of renal sonographic images had been excluded in this study. The procedures were in accordance with the Helsinki Declaration. The study protocol was approved by the Ethics Review Committee of Beijing Luhe Hospital. Written informed consents were obtained from all participants involved in the study.

### Anthropometric measurements and laboratory data

All patients participating in our study underwent a physical examination including height, weight, waist circumference, hip circumference, systolic blood pressures (SBP) and diastolic blood pressures (DBP). The clinical information such as age, gender, duration of diabetes was collected through the medical record of each subject. The venous blood and urine samples were collected in the morning following an overnight fast for the examination of the biochemical indices. The level of SUA, fasting serum glucose (FBG), TC, TG, low-density lipoprotein-cholesterol (LDL-C), high-density lipoprotein-cholesterol (HDL-C), serum creatinine (sCr) and blood urea nitrogen (BUN) were measured by an auto-biochemical analyzer (Roche/Hitachi Cobas C501, Roche Diagnostic Corp., Indianapolis). The glycosylated hemoglobin (HbA1c) was quantified using high-performance liquid chromatography (HPLC) with a D10 set (Bio-RAD, Hercules, California). The ratio of urinary microalbumin to creatinine (UACR) was determined using an early-morning first sterile urine sample with the electrochemical luminescence methods (Roche Diagnostics GmbH, Germany). The sensitivity and coefficient of variation of above parameters were shown in supplementary materials (Table S4). The eGFR was calculated using the Chronic Kidney Disease Epidemiology Collaboration (CKD-EPI) equation. The eGFR = 141 × min (Scr/κ,1) ^α^ × max (Scr/κ, 1) ^−1.209^ × 0.993 ^Age^ × 1.018 [if female] × 1.159 [if black]. (Scr is serum creatinine in µmol/L, κ is 61.9 for females and 79.6 for males, α is -0.329 for females and -0.411 for males, min indicates the minimum of Scr/κ or 1, and max indicates the maximum of Scr/κ or 1). Renal dysfunction was defined as eGFR < 90 ml/(min*1.73m^2^). The BMI was calculated as weight divided by the square of height (kg/m^2^) and was classified into two categories: non-obesity (BMI < 27.5 kg/m^2^) and obesity (BMI ≥ 27.5 kg/m^2^) [26]. The waist-to-hip ratio (WHR) was calculated by dividing waist circumference by hip circumference.

### Measurement of PrFT and PnFT

PrFT and PnFT were detected as previously described by our group [19]. Briefly, PrFT and PnFT were measured by a single skilled operator, using a duplex Doppler apparatus (Model Preirus, HITACHI), with patients in the supine position. The operator was unaware of the clinical data of all subjects. The probe was held vertical to the skin on the lateral aspects of the abdomen to obtain the optimal position. The pressure of the probe on the skin surface was as small as possible to prevent the fat layer from being compressed. The PrFT was determined from the renal fascia to the surface of the kidney. The PnFT was then determined from the inner side of the abdominal musculature to the renal fascia (Figure S1). The PrFT and PnFT were measured three times on both sides. The average of the ultrasound measure on both sides was defined as PrFT and PnFT. The correlation between the bilateral measurements of PrFT and PnFT were shown in supplementary materials (Figures S2 and S3). The intraoperator coefficient of variation was reported to be 4.5% [23].

### Statistics

The statistical analysis was performed using statistical package R (version 3.5.2, available from http://www.r-project.org). Normal distribution of continuous variables was detected using histogram and Q-Q plot. Nearly normally distributed continuous variables were presented as mean ± standard derivation and the differences were compared by student’s t test. UACR, which did not fit nonnormal distribution, was presented as median and quartiles and compared by Mann–Whitney U Test. Categorical variables were presented as frequencies (proportions) and compared by Chi-square test. When the theoretical value < 1, Fisher’s exact test was used. Pearson correlation coefficient was used to assess the relationship between different parameters. Univariate and Multivariate linear regression analysis were used to evaluate the association between the SUA level and other parameters. *P* value < 0.05 was considered statistically significant.

## Results

### Clinical characteristics of the study population

A total of 257 patients with T2DM were enrolled in our study. The mean age was 58.7 ± 14.5 years, and 52.1% were females. The average SUA was 328 ± 94.6 μmol/l. The mean value of PrFT and PnFT were 0.96 ± 0.47 cm and 1.0 ± 0.39 cm respectively. The clinical and the metabolic characteristics of the study population stratified by the tertiles of SUA level were shown in Table 1. The patients with higher SUA level had higher level of PrFT and PnFT than those with lower SUA level (1.10 ± 0.45 cm vs. 0.84 ± 0.49 cm for PrFT, 1.10 ± 0.39 cm vs. 0.92 ± 0.38 cm for PnFT, *P* = 0.001 and *P* = 0.009, respectively). In addition, the age, BMI, WHR, HbA1c, BUN, sCr and UACR in patients with higher SUA level were higher compared to patients with lower SUA level (Table 1). The clinical and the metabolic characteristics of the study population stratified by different renal function, gender and BMI groups were shown in supplementary materials (Table S1, Table S2 and Table S3). The SUA level in patients with normal renal function was slightly lower than that in patient with renal dysfunction (320.69 ± 95.58 umol/l vs. 340.27 ± 92.14 umol/l, *P* = 0.109), but with no statistical significance (Table S1). A significant difference in SUA level was found between males and females (342 ± 92.1 μmol/l vs. 315 ± 95.4 μmol/l respectively, *P* = 0.023). In addition, males had higher PrFT and PnFT than that of females (Table S2). The SUA level in obese patients was obviously higher than that in non-obese patients (363.03 ± 97.05 umol/l vs. 307.07 ± 86.78 umol/l, *P* < 0.001, Table S3).

**Table1:** The general clinical characteristics of the study population stratified by the tertiles of SUA

Parameters	SUA T1 (*n* = 84)	SUA T2 (*n* = 85)	SUA T3 (*n* = 86)	*P* value
Gender (Female%)	51 (60.7)	43 (50.6)	39 (45.3)	0.126
Age (years)	61.98 ± 12.10	58.14 ± 13.88	56.21 ± 16.86	0.031
BMI (kg/ m^2^)	25.19 ± 3.68	26.72 ± 3.67	27.58 ± 4.00	< 0.001
WHR	0.94 ± 0.06	0.95 ± 0.07	0.96 ± 0.06	0.031
Duration (years)	9.98 ± 7.95	10.01 ± 7.69	10.86 ± 8.39	0.722
FBG (mmol/l)	8.25 ± 2.89	7.74 ± 2.21	7.58 ± 2.85	0.246
HbA1c (%)	9.98 ± 2.30	9.27 ± 1.91	8.91 ± 1.95	0.003
SBP (mmHg)	126.73 ± 14.13	130.85 ± 16.00	131.73 ± 16.87	0.089
DBP (mmHg)	73.75 ± 9.85	76.60 ± 11.04	77.83 ± 12.12	0.050
BUN (mmol/l)	5.10 ± 1.52	5.20 ± 1.60	5.79 ± 2.41	0.040
sCr (μmol/l)	64.48 ± 17.85	65.18 ± 18.56	75.62 ± 20.66	< 0.001
eGFR(ml/(min*1.73m^2^))	93.17 ± 17.98	96.82 ± 18.87	90.05 ± 24.96	0.107
UACR (mg/g)	8.1(4.0—25.0)	9.7 (4.7—26.0)	22(5.1—93.0)	0.049
SUA (μmol/l)	229.76 ± 37.56	318.69 ± 24.41	433.56 ± 62.19	< 0.001
PrFT (cm)	0.84 ± 0.49	0.95 ± 0.42	1.10 ± 0.45	0.001
PnFT (cm)	0.92 ± 0.38	0.97 ± 0.38	1.10 ± 0.39	0.009
TC (mmol/l)	4.16 ± 1.13	4.42 ± 1.28	4.54 ± 1.48	0.159
TG (mmol/l)	1.50 ± 1.17	1.62 ± 0.74	2.06 ± 1.49	0.006
HDL-c (mmol/l)	1.11 ± 0.28	1.06 ± 0.25	1.02 ± 0.24	0.075
LDL-c (mmol/l)	2.62 ± 0.84	2.93 ± 1.04	2.97 ± 1.12	0.050
Smoke (%)	30 (35.7)	33 (38.8)	33 (38.4)	0.903
Drink (%)	14 (16.7)	25 (29.4)	24 (27.9)	0.111
Hypertension (%)	54 (64.3)	49 (57.6)	56 (65.1)	0.545
Coronary heart disease (%)	26 (31.0)	20 (23.5)	21 (24.4)	0.489

UACR were presented as median and quartile

*BMI* Body Mass Index, *WHR* Waist-to-hip Ratio, *Duration* Duration of diabetes mellitus, *FBG* Fasting Blood Glucose, *HbA1c* glycosylated Hemoglobin, *SBP* Systolic Blood Pressure, *DBP* Diastolic Blood Pressure, *BUN* Blood Urea Nitrogen, *sCr* serum Creatinine, *eGFR* estimated Glomerular Filtration Rate, *UACR* the ratio of Urinary Albumin to Creatinine, *SUA* Serum Uric Acid, *PrFT* Perirenal Fat Thickness, *PnFT* Paranephric Fat Thickness, *TC* Total Cholesterol, *TG* Triglyceride, *HDL-c* High Density Lipoprotein-cholesterol, *LDL-c* Low Density Lipoprotein-cholesterol

^*^P values < 0.05

### Association of other parameters with SUA level in all patients

We used the Pearson correlation coefficient to assess the relationship between SUA level and other parameters. Table 2 showed the correlation of SUA with all other parameters. The PrFT and PnFT were positively associated with SUA level (*r* = 0.25, *P* < 0.001, *r* = 0.23, *P* < 0.001, respectively). In addition, the BMI, WHR, BNU, sCr, TC, TG and LDL-c were significantly and positively associated with SUA level. While age, HbA1c and HDL-c were negatively correlated with SUA level. The visualization of correlation matrix was shown in supplementary Figure S4.

**Table 2:** Association of SUA level with all other investigated parameters in all patients

Parameters	r	*P* value
Age (years)	- 0.19	0.002
BMI (kg/m^2^)	0.28	< 0.001
WHR	0.13	0.033
PrFT (cm)	0.25	< 0.001
PnFT (cm)	0.23	< 0.001
Duration (year)	0.01	0.921
FBG (mmol/l)	- 0.06	0.347
HbA1c (%)	- 0.17	0.006
SBP (mmHg)	0.17	0.238
DBP (mmHg)	0.12	0.053
BUN (mmol/l)	0.17	0.008
Cr (μmol/l)	0.31	< 0.001
eGFR(ml/(min*1.73m^2^))	- 0.10	0.129
Log (UACR)	0.18	0.004
TC (mmol/l)	0.15	0.019
TG (mmol/l)	0.22	< 0.001*
HDL-c (mmol/l)	- 0.14	0.025*
LDL-c (mmol/l)	0.18	0.004*

*BMI* Body Mass Index, *WHR* Waist-to-hip Ratio, *Duration* Duration of diabetes mellitus, *FBG* Fasting Blood Glucose, *HbA1c* glycosylated Hemoglobin, *SBP* Systolic Blood Pressure, *DBP* Diastolic Blood Pressure, *BUN* Blood Urea Nitrogen, *Cr* serum Creatinine, *eGFR* estimated Glomerular Filtration Rate, *UACR* the ratio of Urinary Albumin to Creatinine, *PrFT* Perirenal Fat Thickness, *PnFT* Paranephric Fat Thickness, *TC* Total Cholesterol, *TG* Triglyceride, *HDL-c* High Density Lipoprotein-cholesterol, *LDL-c* Low Density Lipoprotein-cholesterol

^*^P values < 0.05

### Association of PrFT and PnFT with SUA level in different subgroups

**Fig. 1:**
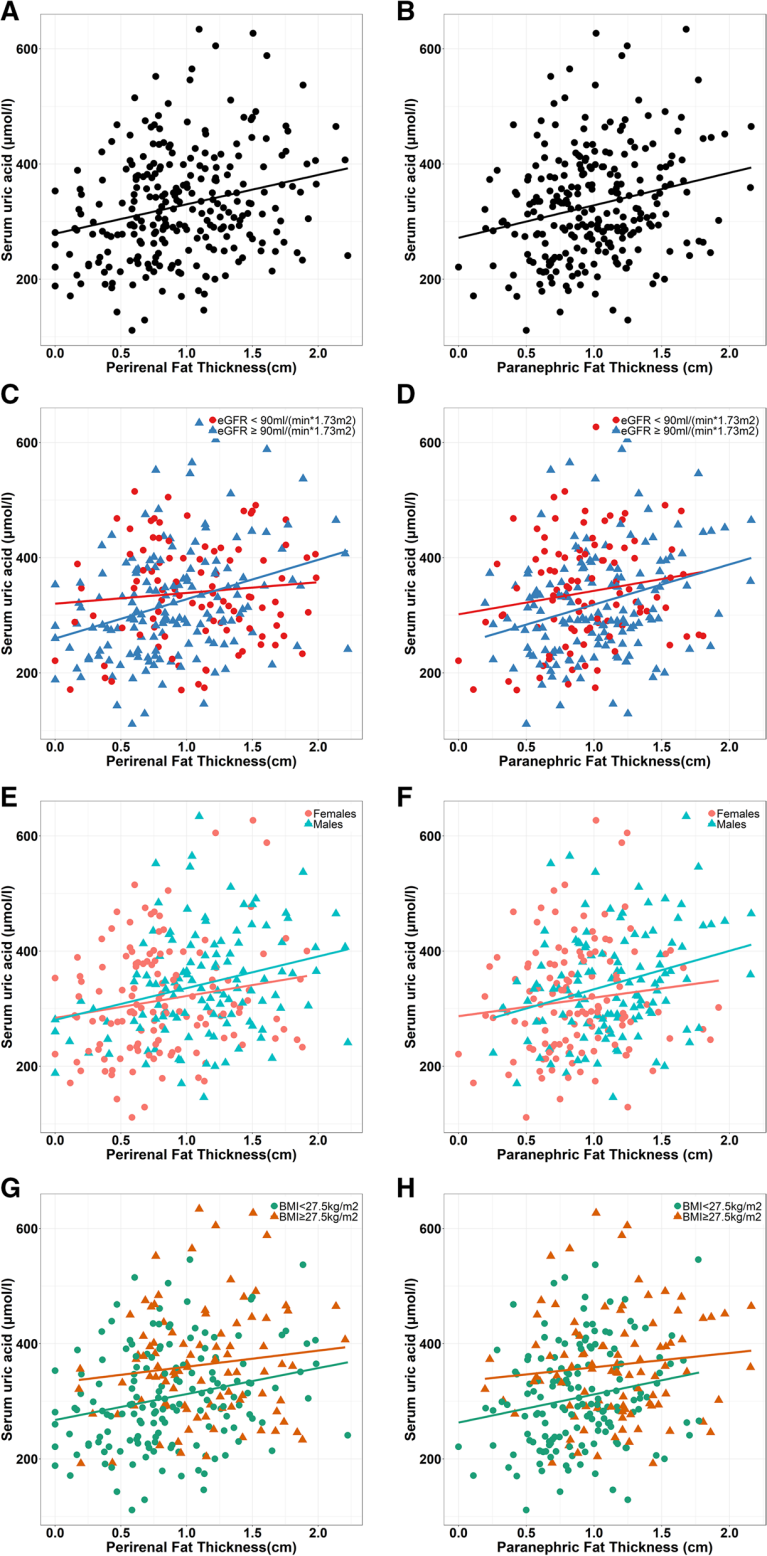
Association of PrFT and PnFT with SUA in different subgroups. **A** Association between PrFT and SUA. **B** Association between PnFT and SUA. **C** Association between PrFT and SUA in different renal function. **D** Association between PnFT and SUA in different renal function. **E** Association between PrFT and SUA in gender subgroups. **F** Association between PnFT and SUA in gender subgroups. **G**. Association between PrFT and SUA in BMI subgroups. **H** Association between PnFT and SUA in BMI subgroups PrFT, perirenal fat thickness, PnFT, paranephric fat thickness, SUA, serum uric acid

Considering the potential effect of multiple factors such as gender, BMI and other indices on SUA level. We stratified all subjects into different groups according to renal function (eGFR ≥ 90 ml/(min*1.73m^2^) and eGFR < 90 ml/(min*1.73m^2^)), gender (females and males) and BMI (BMI < 27.5 kg/m^2^ and BMI ≥ 27.5 kg/m^2^). We further conducted Pearson correlation analysis in different subgroups. Results were shown in Table 3 and Fig. 1. We found that the PrFT was significantly positively associated with SUA level (*r* = 0.25, *P* < 0.001). Moreover, the relationship between PrFT and SUA became obvious in males (*r* = 0.27, *P* = 0.003) and patients with normal renal function (*r* = 0.32, *P* < 0.001). In addition, PrFT was significantly associated with SUA level in non-obese patients (*r* = 0.24, *P* = 0.002) but not in obese patients (*r* = 0.12, *P* = 0.226). The PnFT was also positively associated with SUA level (*r* = 0.23, *P* < 0.001). This relationship became obvious in males (*r* = 0.28, *P* = 0.002) and patients with normal renal function (*r* = 0.29, *P* < 0.001) but not in females (*r* = 0.12, *P* = 0.177) and patients with renal dysfunction (*r* = 0.16, *P* = 0.108). In addition, PnFT was significantly associated with SUA level in non-obese patients (*r* = 0.19, *P* = 0.015) but not in obese patients (*r* = 0.10, *P* = 0.311).

**Table 3:** Association of PrFT and PnFT with SUA level in different groups

Subgroups	PrFT		PnFT	
	r	*P* value	r	*P* value
All patients	0.25	< 0.001	0.23	< 0.001
Renal function				
eGFR ≥ 90 ml/(min*1.73m^2^)	0.32	< 0.001	0.29	< 0.001
eGFR < 90 ml/(min*1.73m^2^)	0.09	0.359	0.16	0.108
Gender				
Females	0.17	0.047	0.12	0.177
Males	0.27	0.003	0.28	0.002
BMI				
Non-obesity (BMI < 27.5 kg/m^2^)	0.24	0.002	0.19	0.015
Obesity (BMI ≥ 27.5 kg/m^2^)	0.12	0.226	0.10	0.311

*eGFR* estimated Glomerular Filtration Rate

Non-obesity, BMI < 27.5 kg/m^2^, Obesity, BMI ≥ 27.5 kg/m^2^

^*^P values < 0.05

### Multivariate analysis for the association of PrFT and PnFT with SUA

To further confirm the relationship between PrFT and PnFT and SUA level, we performed multivariate liner regression model. Variance inflation factor (VIF) was calculated to evaluate the collinearity of variables in models. The VIF results showed that there was no existence collinearity in all models (data not show). In the Model 1, both the PrFT and PnFT were positively correlated with SUA level (β = 51.01, *P* < 0.001, β = 56.46, *P* < 0.001, respectively, Table 4). In the Model 2, after adjusting age and gender, both the PrFT and PnFT were still significantly correlated to SUA level (β = 53.47, *P* < 0.001, β = 44.64, *P* < 0.001, respectively, Table 4). In the Model 3 with age, gender, eGFR, drink, BMI, HbA1c, FBG, LDL-c, SBP and DBP adjusted, the PrFT was still independently correlated to SUA level (β = 33.33, *P* = 0.017, Table 4), while the PnFT was not correlated to SUA level (β = 14.80, *P* = 0.378, Table 4).

**Table 4:** Multivariate linear regression analysis for the association of parameters and SUA in all patients

Parameters	β	*P* value
Model 1		
PrFT (cm)	51.01	< 0.001
PnFT (cm)	56.46	< 0.001
Model 2		
PrFT (cm)	53.47	< 0.001
PnFT (cm)	44.64	< 0.001
Model 3		
PrFT (cm)	33.33	0.017
PnFT (cm)	14.80	0.378

*PrFT* Perirenal Fat Thickness, *PnFT* Paranephric Fat Thickness

Model 1 adjusting no variables

Model 2 adjusting for age and gender

Model 3 adjusting for age, gender, eGFR, drink, BMI, HbA1c, FBG, LDL-c, SBP and DBP

^*^P values < 0.05

## Discussion

We conducted a cross-sectional study to explore the relationship between PrFT and PnFT and SUA level in T2DM patients. The main finding of our study was that the PrFT was independently and positively associated with SUA level in patients with T2DM. As previous studies reported, PrFT and PnFT were positively associated with SUA respectively in diabetic patients [19, 24], while some confounders were not adjusted. Similarly, Lamacchia et al. and Giulio et al. reported that para-and perirenal fat thickness was significantly and positively associated with SUA in diabetic patients and hypertensive patients respectively [23, 25]. However, PrFT was not distinguished from PnFT in the above studies. As mentioned in the background, perirenal fat was different from paranephric fat histologically and physiologically. So, we explored the relationship between PrFT and PnFT and SUA separately and demonstrated that the PrFT was independently and positively associated with SUA level after adjusting age, gender, eGFR, drink, BMI, HbA1c, FBG, LDL-c, SBP and DBP. While PnFT, in present study, was not significantly associated with SUA after adjusting confounders.

Moreover, we found that the level of SUA, PrFT and PnFT in males were higher than that in females, which was consistent with previous studies [1, 27–29]. In the subgroup analysis, Pearson correlation analysis showed that the relationship between PrFT and PnFT and SUA in males was stronger than that in females. While another study conducted by Guo et al. presented a higher correlation between PrFT and SUA in females [24].That study enrolled patients with newly diagnosed T2DM and with mean age of 52.5 ± 8.2 years, which may explain the contrary results. In addition, we also found that the relationship between PrFT and PnFT and SUA was not significant in patients with reduced renal function. It was reported that PrFT was independently and positively associated with renal function [18, 19]. This finding indicates that the effect of PrFT on SUA may be independent on reduced renal function. More studies about the mechanism between PrFT, PnFT, SUA and renal function were needed in the future.

Pearson correlation analysis also showed that TC, TG and LDL-c were positively associated with SUA level and HDL-c was negatively correlated with SUA level, which was concordant with previous studies [13, 27]. While HbA1c was negatively correlated with SUA level in our study. A growing body of evidence has pointed out that the relationship between HbA1c and SUA was affected by gender and glucose level [30–32]. Previous studies have shown that HbA1c was negatively associated with SUA level in males, but positively associated with SUA level in females [30, 31]. Wei et al. indicated that HbA1c was positively correlated with SUA in subjects with normal glucose level but negatively correlated with SUA in T2DM patients [32]. Decreased SUA level may be caused by increased renal excretion of UA in the presence of hyperglycemia.

Several hypotheses about the mechanism by which how PrFT affected the SUA level were proposed. First, when perirenal fat grows into the renal sinus, various renal structures, including the medullary vasa recta and tubules, can be compressed by the increased renal interstitial fluid hydrostatic pressure, reducing blood and tubular flow through the distensible loop of Henle. The decreased tubular transit velocity and medullary blood flow may likely promote uric acid reabsorption. These findings may likely provide an explanation for the gradual increase of SUA level [25]. In addition, excessive free fatty acids released from perirenal fat may lead to renal lipotoxicity by both endocrine and paracrine pathways. Third, previous animal experiments have reported that perirenal fat damaged renal vascular endothelial dysfunction through increasing oxidative stress and activating inflammatory molecular pathways [33, 34].

Our study had several limitations. First of all, this study did not assess different lifestyles, dietary habits, which may influence the SUA level. In addition, the renal resistive index, which can reflect the hemodynamic characteristics and renal function, was not calculated in our study. Furthermore, in our study, the measure of PrFT and PnFT was not validated with computed tomography, while previous studies had reported that ultrasonography and computed tomography have a good correlation in the measure of PrFT and PnFT [35, 36].

In conclusion, PrFT was independently and positively associated with SUA level in patients with T2DM. This indicated that PrFT maybe an important indicator of hyperuricemia in patients with T2DM. In clinical practice, the measurement of perirenal fat may be helpful to find the population with high risk of hyperuricemia. Moreover, reducing the mass of perirenal fat maybe a new therapy for the treatment of hyperuricemia.

## Supplementary Information

**Additional file 1: Table S1.** The clinical characteristics of the study population in different renal function. **Table S2.** The clinical characteristics of the study population in different genders. **Table S3.** The clinical characteristics of the study population in different BMI groups. **Table S4.** The sensitivity and coefficient of variation of parameters in the study. **Figure S1.** Ultrasound image of PrFT and PnFT. **Figure S2.** The correlation between the left PrFT and right PrFT. **Figure S3.** The correlation between the left PnFT and right PnFT. **Figure S4.** The visualization of correlation matrix between PrFT and PnFT and other Parameters in patients with type 2 diabetes mellitus.

## Data Availability

The datasets analyzed during the current study are not publicly available due to some incomplete work of our team but are available from the corresponding author on reasonable request.
