# Supplementary material for: The perirenal fat thickness was independently associated with serum uric acid level in patients with type 2 diabetes mellitus
Source: BMC Endocr Disord. 2022 Aug 20;22:210. doi: 10.1186/s12902-022-01081-9 (PMC9392942; doi:10.1186/s12902-022-01081-9)
Supplement: Supplementary file 1 — Additional file 1: Table S1. The clinical characteristics of the study population in different renal function. Table S2. The clinical characteristics of the study population in different genders. Table S3. The clinical characteristics of the study population in different BMI groups. Table S4. The sensitivity and coefficient of variation of parameters in the study. Figure S1. Ultrasound image of PrFT and PnFT. Figure S2. The correlation between the left PrFT and right PrFT. Figure S3. The correlation between the left PnFT and right PnFT. Figure S4. The visualization of correlation matrix between PrFT and PnFT and other Parameters in patients with type 2 diabetes mellitus. [file 12902_2022_1081_MOESM1_ESM.doc]

**Table S1. The clinical characteristics of the study population in different renal function.**

| Parameters | eGFR ≥ 90ml/(min*1.73m^2^)  (n=159) | eGFR < 90ml/(min*1.73m^2^)  (n=97) | *P* values |
| --- | --- | --- | --- |
| Gender (Female%) | 79 (49.7) | 54 (55.7) | 0.354 |
| Age (years) | 52.00 ± 13.36 | 69.74 ± 8.45 | <0.001 |
| BMI (kg/ m^2^) | 26.92 ± 4.03 | 25.82 ± 3.57 | 0.028 |
| WHR | 0.95 ± 0.06 | 0.96 ± 0.07 | 0.106 |
| Duration (years) | 7.96 ± 6.75 | 14.10 ± 8.39 | <0.001 |
| FBG (mmol/l) | 8.01 ± 2.63 | 7.61 ± 2.74 | 0.259 |
| HbA1c (%) | 9.53 ± 1.97 | 9.10 ± 2.30 | 0.114 |
| SBP (mmHg) | 128.35 ± 15.83 | 132.15 ± 15.48 | 0.061 |
| DBP (mmHg) | 78.29 ± 10.89 | 72.51 ± 10.62 | <0.001 |
| BUN (mmol/l) | 4.64 ± 1.28 | 6.60 ± 2.16 | <0.001 |
| sCr (μmol/l) | 59.14 ± 12.17 | 83.87 ± 19.96 | <0.001 |
| eGFR(ml/(min*1.73m^2^)) | 106.17 ± 11.72 | 72.33 ± 14.79 | <0.001 |
| UACR (mg/g) | 8.2 (3.9 - 26.0) | 16.7 (5.6-104.0) | 0.001 |
| SUA (μmol/l) | 320.69 ± 95.58 | 340.27 ± 92.14 | 0.109 |
| PrFT (cm) | 0.90 ± 0.45 | 1.08 ± 0.47 | 0.002 |
| PnFT (cm) | 1.03 ± 0.40 | 0.94 ± 0.37 | 0.083 |
| TC (mmol/l) | 4.52 ± 1.38 | 4.14 ± 1.15 | 0.028 |
| TG (mmol/l) | 1.88 ± 1.39 | 1.50 ± 0.73 | 0.014 |
| HDL-c (mmol/l) | 1.07 ± 0.26 | 1.05 ± 0.25 | 0.499 |
| LDL-c (mmol/l) | 2.94 ± 1.06 | 2.67 ± 0.91 | 0.038 |
| Smoke (%) | 65 (40.9) | 32 (33.0) | 0.259 |
| Drink (%) | 43 (27.0) | 21 (21.6) | 0.413 |
| Hypertension (%) | 80 (50.3) | 79 (81.4) | <0.001 |
| Coronary heart disease (%) | 28 (17.6) | 39 (40.2) | <0.001 |

UACR were presented as median and quartile.

BMI, body mass index, WHR, waist-to-hip ratio, Duration, duration of diabetes mellitus, FBG, fasting blood glucose,HbA1c, glycosylated hemoglobin ,SBP, systolic blood pressure, DBP, diastolic blood pressure, BUN, blood urea nitrogen, sCr, serum creatinine, eGFR, estimated glomerular filtration rate, UACR, the ratio of urinary albumin to creatinine, SUA, serum uric acid, PrFT, perirenal fat thickness, PnFT, paranephric fat thickness, TC, total cholesterol, TG, triglyceride, HDL-c, high density lipoprotein-cholesterol, LDL-c, low density lipoprotein-cholesterol.

**Table S2. The clinical characteristics of the study population in different genders.**

| Parameters | Males  (n=123) | Females  (n=134) | *P* values |
| --- | --- | --- | --- |
| Age (years) | 55.60 ± 14.99 | 61.51 ± 13.57 | 0.001 |
| BMI (kg/ m^2^) | 26.81 ± 3.80 | 26.17 ± 3.99 | 0.188 |
| WHR | 0.96 ± 0.06 | 0.94 ± 0.05 | 0.001 |
| Duration (years) | 8.78 ± 7.39 | 11.71 ± 8.25 | 0.003 |
| FBG (mmol/l) | 7.98 ± 2.85 | 7.74 ± 2.51 | 0.490 |
| HbA1c (%) | 9.16 ± 2.04 | 9.58 ± 2.16 | 0.110 |
| SBP (mmHg) | 130.41 ± 16.27 | 129.12 ± 15.33 | 0.512 |
| DBP (mmHg) | 78.44 ± 10.66 | 73.82 ± 11.19 | 0.001 |
| BUN (mmol/l) | 5.56 ± 1.82 | 5.20 ± 1.99 | 0.131 |
| sCr (μmol/l) | 76.76 ± 17.83 | 60.88 ± 18.17 | <0.001 |
| eGFR(ml/(min*1.73m^2^)) | 95.67 ± 20.58 | 91.2 ± 21.09 | 0.088 |
| UACR (mg/g) | 9 (4.4-44) | 10.9 (4.5-37) | 0.936 |
| SUA (μmol/l) | 342 ± 92.1 | 315 ± 95.4 | 0.023 |
| PrFT (cm) | 1.12 ± 0.45 | 0.82 ± 0.43 | <0.001 |
| PnFT (cm) | 1.13 ± 0.39 | 0.88 ± 0.35 | <0.001 |
| TC (mmol/l) | 4.39 ± 1.36 | 4.36 ± 1.27 | 0.854 |
| TG (mmol/l) | 1.90 ± 1.41 | 1.58 ± 0.95 | 0.034 |
| HDL-c (mmol/l) | 1.01 ± 0.25 | 1.11 ± 0.26 | 0.002 |
| LDL-c (mmol/l) | 2.88 ± 0.99 | 2.80 ± 1.04 | 0.520 |
| Smoke (%) | 90 (73.2) | 7 (5.2) | <0.001 |
| Drink (%) | 59 (48.0) | 5 (3.7) | <0.001 |
| Hypertension (%) | 73 (59.3) | 86 (64.2) | 0.504 |
| Coronary heart disease (%) | 27 (22.0) | 40 (29.9) | 0.194 |

UACR were presented as median and quartile.

BMI, body mass index, WHR, waist-to-hip ratio, Duration, duration of diabetes mellitus, FBG, fasting blood glucose,HbA1c, glycosylated hemoglobin ,SBP, systolic blood pressure, DBP, diastolic blood pressure, BUN, blood urea nitrogen, sCr, serum creatinine, eGFR, estimated glomerular filtration rate, UACR, the ratio of urinary albumin to creatinine, SUA, serum uric acid, PrFT, perirenal fat thickness, PnFT, paranephric fat thickness, TC, total cholesterol, TG, triglyceride, HDL-c, high density lipoprotein-cholesterol, LDL-c, low density lipoprotein-cholesterol.

**Table S3. The clinical characteristics of the study population in different BMI groups.**

UACR were presented as median and quartile. Non-obesity, BMI < 27.5 kg/m2, Obesity, BMI ≥ 27.5 kg/m2.

| Parameters | Non-obesity  (n=161) | Obesity  (n=96) | *P* values |
| --- | --- | --- | --- |
| Gender (Female%) | 89 (55.3) | 45 (46.9) | 0.240 |
| Age (years) | 61.84 ± 12.65 | 53.38 ± 15.96 | <0.001 |
| BMI (kg/ m^2^) | 24.06 ± 2.30 | 30.54 ± 2.34 | <0.001 |
| WHR | 0.94 ± 0.06 | 0.97 ± 0.06 | <0.001 |
| Duration (years) | 11.06 ± 7.75 | 9.04 ± 8.21 | 0.049 |
| FBG (mmol/l) | 7.70 ± 2.67 | 8.12 ± 2.68 | 0.230 |
| HbA1c (%) | 9.49 ± 2.27 | 9.20 ± 1.82 | 0.299 |
| SBP (mmHg) | 128.61 ± 16.59 | 131.62 ± 14.17 | 0.139 |
| DBP (mmHg) | 73.71 ± 11.02 | 79.93 ± 10.31 | <0.001 |
| BUN (mmol/l) | 5.53 ± 1.97 | 5.13 ± 1.80 | 0.108 |
| sCr (μmol/l) | 68.35 ± 20.49 | 68.78 ± 18.27 | 0.865 |
| eGFR (ml/(min*1.73m^2^)) | 90.80 ± 19.64 | 97.60 ± 22.37 | 0.012 |
| UACR (mg/g) | 7.5 (3.9-28.0) | 15 (5.6-56.0) | 0.013 |
| SUA (μmol/l) | 307.07 ± 86.78 | 363.03 ± 97.05 | <0.001 |
| PrFT (cm) | 0.88 ± 0.47 | 1.11 ± 0.43 | <0.001 |
| PnFT (cm) | 0.90 ± 0.34 | 1.16 ± 0.41 | <0.001 |
| TC (mmol/l) | 4.27 ± 1.21 | 4.55 ± 1.46 | 0.101 |
| TG (mmol/l) | 1.56 ± 1.12 | 2.02 ± 1.28 | 0.003 |
| HDL-c (mmol/l) | 1.09 ± 0.26 | 1.01 ± 0.25 | 0.017 |
| LDL-c (mmol/l) | 2.73 ± 0.94 | 3.02 ± 1.11 | 0.025 |
| Smoke (%) | 56 (34.8) | 41 (42.7) | 0.256 |
| Drink (%) | 38 (23.6) | 26 (27.1) | 0.635 |
| Hypertension (%) | 101 (62.7) | 58 (60.4) | 0.813 |
| Coronary heart disease (%) | 45 (28.0) | 22 (22.9) | 0.458 |

BMI, body mass index, WHR, waist-to-hip ratio, Duration, duration of diabetes mellitus, FBG, fasting blood glucose,HbA1c, glycosylated hemoglobin ,SBP, systolic blood pressure, DBP, diastolic blood pressure, BUN, blood urea nitrogen, sCr, serum creatinine, eGFR, estimated glomerular filtration rate, UACR, the ratio of urinary albumin to creatinine, SUA, serum uric acid, PrFT, perirenal fat thickness, PnFT, paranephric fat thickness, TC, total cholesterol, TG, triglyceride, HDL-c, high density lipoprotein-cholesterol, LDL-c, low density lipoprotein-cholesterol.

**Table S4.** **The sensitivity and coefficient of variation of parameters in the study.**

| Parameters | Sensitivity | Coefficient of variation (%) |
| --- | --- | --- |
| FBG (mmol/l) | 0.2 | <5 |
| HbA1c (%) | 3 | <2 |
| BUN (mmol/l) | 0.17 | <5 |
| sCr (μmol/l) | 30 | <5 |
| SUA (μmol/l) | 29 | <4 |
| TC (mmol/l) | 0.3 | <4 |
| TG (mmol/l) | 0.3 | <5 |
| HDL-c (mmol/l) | 0.2 | <4 |
| LDL-c (mmol/l) | 0.3 | <3 |

FBG, fasting blood glucose, HbA1c, glycosylated hemoglobin, BUN, blood urea nitrogen, sCr, serum creatinine, SUA, serum uric acid, TC, total cholesterol, TG, triglyceride, HDL-c, high density lipoprotein-cholesterol, LDL-c, low density lipoprotein-cholesterol.

**
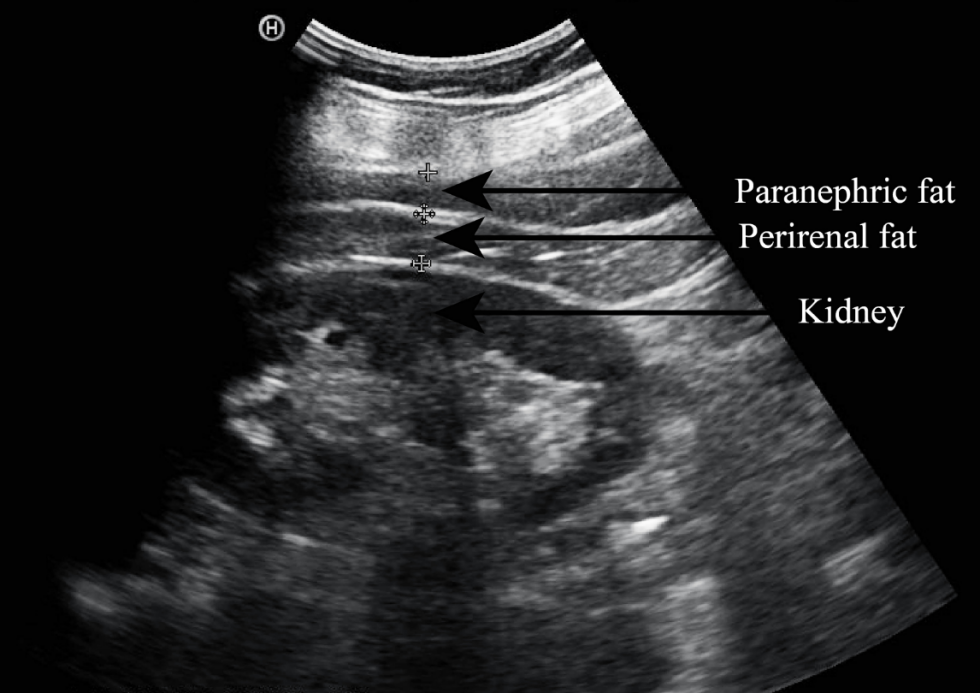
**

**Figure S1. Ultrasound image of Perirenal fat thickness and paranephric fat thickness.**

The PrFT was determined from the renal fascia to the surface of the kidney. The PnFT was then determined from the inner side of the abdominal musculature to the renal fascia.


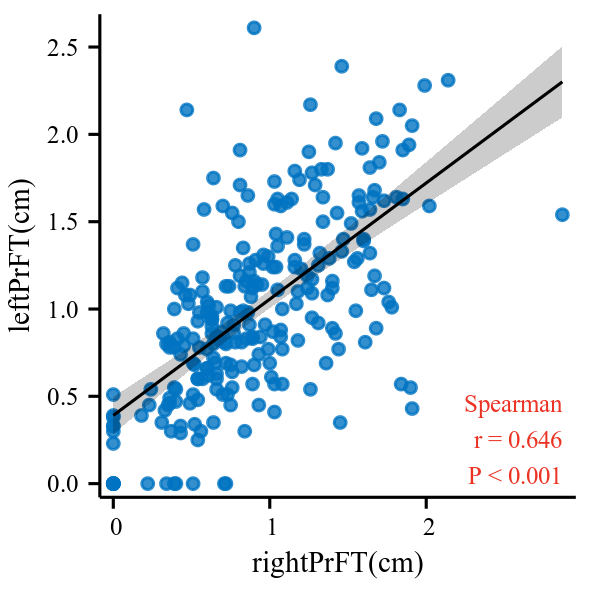


**Figure S2. The correlation between the leftPrFT and rightPrFT.**


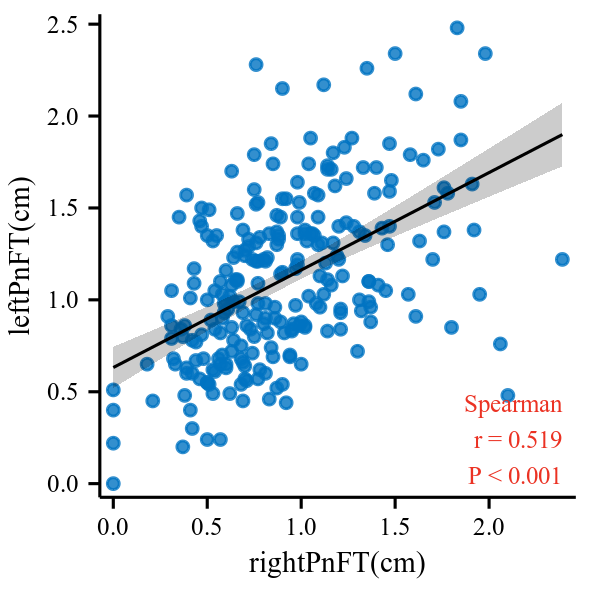


**Figure S3. The correlation between the leftPnFT and rightPnFT.**


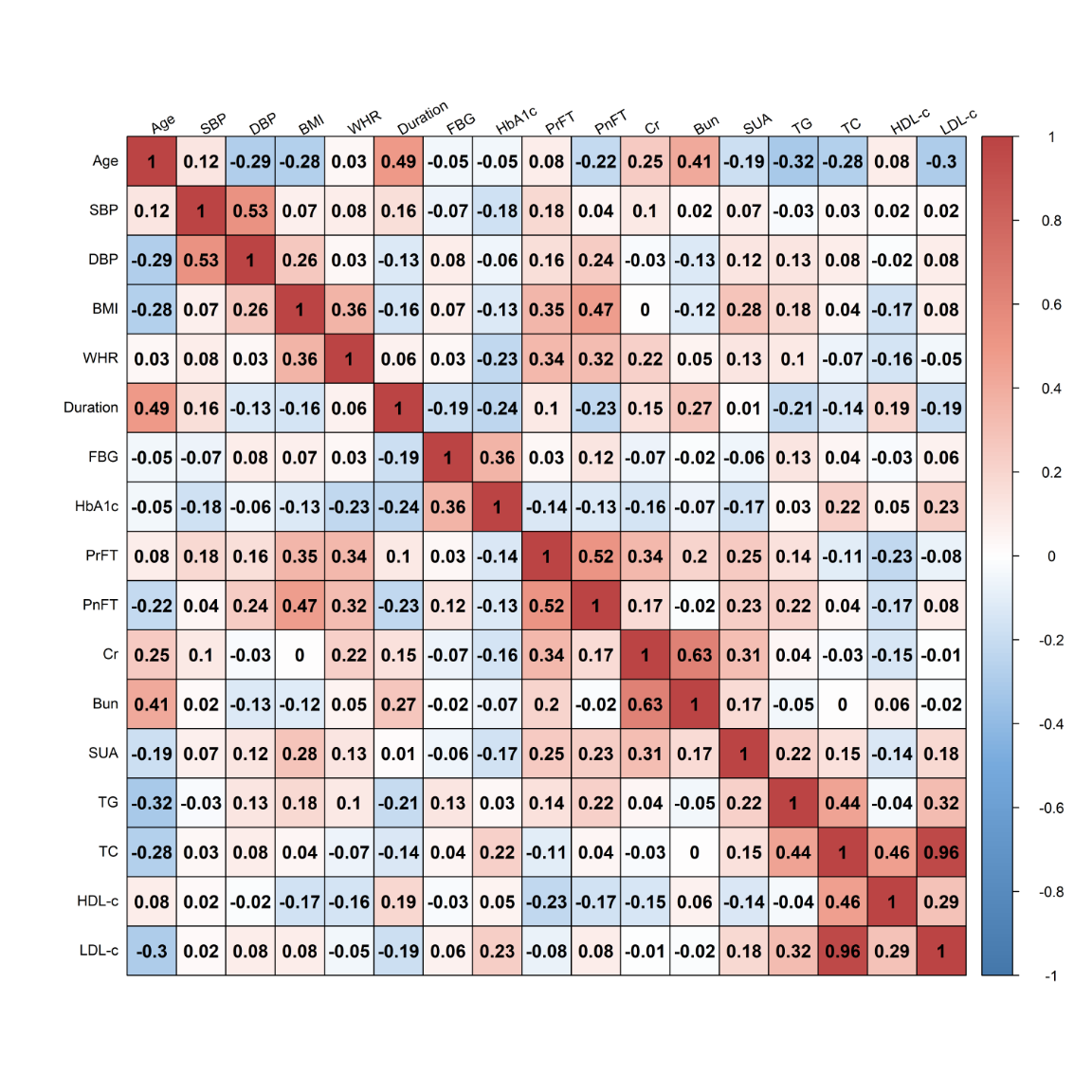


**Figure S4. The visualization of correlation matrix between perirenal fat thickness and paranephric fat thickness and other Parameters in patients with type 2 diabetes mellitus**

SBP, systolic blood pressure, DBP, diastolic blood pressure, BMI, body mass index, WHR, waist-to-hip ratio, Duration, duration of diabetes mellitus, FBG, fasting blood glucose, HbA1c, glycosylated hemoglobin, PrFT, perirenal fat thickness, PnFT, paranephric fat thickness, Cr, creatinine, BUN, blood urea nitrogen, SUA, serum uric acid, TG, triglyceride, TC, total cholesterol, HDL-c, high density lipoprotein-cholesterol, LDL-c, low density lipoprotein-cholesterol.
